# Dexamethasone Modulates the Dynamics of Wnt Signaling in Human Trabecular Meshwork Cells

**DOI:** 10.3390/vision7020043

**Published:** 2023-05-25

**Authors:** Chi Zhang, Elizabeth Tannous, Alseena Thomas, Natalia Jung, Edmond Ma, Jie J. Zheng

**Affiliations:** Stein Eye Institute, Department of Ophthalmology, David Geffen School of Medicine, The Molecular Biology Institute at the University of California, Los Angeles, CA 90095, USA

**Keywords:** trabecular meshwork, Wnt signaling, myocilin, glaucoma

## Abstract

Trabecular meshwork (TM) tissue is highly specialized, and its structural integrity is crucial for maintaining homeostatic intraocular pressure (IOP). The administration of glucocorticoids, such as dexamethasone (DEX), can perturb the TM structure and significantly increase IOP in susceptible individuals, resulting in ocular diseases such as steroid-induced glaucoma, a form of open-angle glaucoma. Although the exact mechanism involved in steroid-induced glaucoma remains elusive, increasing evidence suggests that DEX may act through various signaling cascades in TM cells. Despite uncertainty surrounding the specific process by which steroid-induced glaucoma occurs, there is growing evidence to indicate that DEX can impact multiple signaling pathways within TM cells. In this study, we examined the impact of DEX treatment on the Wnt signaling pathway in TM cells, given that Wnt signaling has been reported to play a crucial role in regulating extracellular matrix (ECM) levels in the TM. To further elucidate the role of Wnt signaling in the glaucomatous phenotype, we examined mRNA expression patterns between Wnt signaling markers *AXIN2* and *sFRP1* and DEX-mediated induction of myocilin (*MYOC*) mRNA and protein levels over 10 days in DEX-treated primary TM cells. We observed a sequential pattern of peak expression between *AXIN2*, *sFRP1*, and *MYOC.* Based on the study, we propose that *sFRP1* upregulation could be a result of a negative feedback mechanism generated by stressed TM cells to suppress abnormal Wnt signaling activities.

## 1. Introduction

Glaucoma, a collection of diseases that damages the eye’s optic nerve and results in permanent vision loss, is one of the leading causes of blindness in the United States [1,2,3]. Increased intraocular pressure (IOP) is a significant risk factor for glaucoma [4,5,6,7]. IOP is determined by the balance of aqueous humor secreted by the ciliary epithelium and aqueous humor outflow through the conventional pathway involving the trabecular meshwork (TM) and Schlemm’s canal [8]. IOP is elevated when there is greater resistance to aqueous humor outflow located in the inner wall of Schlemm’s canal and the juxtacanalicular region of the TM [9,10].

The administration of glucocorticoids for therapeutic purposes often results in elevated IOP [11,12,13], which can potentially harm the optic nerve, resulting in steroid-induced glaucoma [5]. This is supported by a study conducted in the 1960s, where healthy participants received steroid drops in one eye three times daily for a duration of a month. The study revealed three different types of responders among the participants: the low-response group, which saw an IOP increase of <5 mm Hg in around 66% of the individuals; the intermediate-response group, which experienced an increased IOP of 6 to 15 mm Hg in approximately 30% of volunteers; and the high-response group, which showed an increase of more than 15 mm Hg in about 5% of the volunteers. Furthermore, the IOP increase in the low-response group was observed for 2 weeks before leveling off, while the intermediate- and high-response groups experienced a continuous increase in IOP for 4 weeks [12]. These individuals, accounting for 35% of the total, are referred to as “steroid responders” today. The reason for the elevation in pressure in response to steroid use is due to an increased resistance to the outflow of aqueous humor in the conventional pathway [14,15,16]. Of note, steroid-induced glaucoma and primary open-angle glaucoma (POAG), which is a prevalent form of glaucoma, exhibit a clinical resemblance. Individuals who experience an increase in IOP due to steroid treatment have a notably greater risk of developing POAG than those who do not experience a steroid-induced increase in IOP [17]. These clinical findings suggest a shared pathogenesis mechanism between steroid-induced glaucoma and POAG [15,16,18].

Although the exact mechanism of action remains unknown [7], the pathogenesis mechanism linked to an increased IOP involves the trabecular meshwork [18,19]. The effects of glucocorticoids, such as dexamethasone (DEX), on TM cells have been used as an in vitro model to gain further insight into the biological and molecular signaling consequences that occur in the TM during glaucomatous insult [14,20,21]. TM tissue is highly specialized to filter the aqueous humor before draining into Schlemm’s canal [8,22,23,24]. The structural integrity of the TM is essential for sustaining the equilibrium between aqueous humor production and outflow and in sustaining a physiological IOP [25,26]. TM cells contain glucocorticoid receptors, which are involved in an array of signaling pathways [6,27], and glucocorticoids, such as DEX, may cause changes to the extracellular matrix (ECM) in the TM tissue, resulting in changes to the IOP [7,28]. Cellular signaling cascades in TM cells have been shown to be affected by DEX, some of which may cause abnormalities that are associated with the observed ultrastructural changes to the TM [6,29,30,31]. Indeed, DEX treatment has been linked to drastic changes to the cytoskeleton structure, cross-linked actin networks, and augmented ECM deposition, resulting in morphological changes in components of the TM, which have been implicated in the occlusion of outflow facility and elevated IOP [32,33,34,35].

One well-studied signaling pathway associated with the glaucoma pathology is the Wnt signaling pathway [36,37,38]. Wnt signaling plays a key role in regulating cell fate determination during embryonic development and also plays a critical role in maintaining tissue homeostasis, including HTM [39,40,41,42]. In the current model of the canonical Wnt pathway, secreted Wnt proteins bind the receptor known as Frizzled and lipoprotein-receptor-related protein 5/6, thereby activating Dishevelled (Dvl) proteins inside the cell [43,44]. Dvl proteins then bind to the carboxyl (C)-terminus of the Frizzled receptor [45,46] and recruit Axin [47], a protein member of the β-catenin destruction complex, away from the so-called destruction complex and to the cell membrane. Axin also plays an important role in bringing β-catenin and GSK-3β together so that the phosphorylation and subsequent β-catenin can occur in states where there is no Wnt agonist available [48]. The destruction complex consists of Axin, glycogen synthase kinase-3β (GSK-3β) [49], adenomatous polyposis coli (APC), casein kinase 1α (CK1α), and protein phosphatase 2A (PP2A) [41,50,51]. Without Wnt stimulation, β-catenin is phosphorylated by the GSK-3β in the destruction complex, and the phosphorylated β-catenin can be degraded through the ubiquitin–proteasome system. When Wnt stimulation occurs and activated Dvl proteins recruit Axin to the membrane, GSK-3β is unable to phosphorylate β-catenin and prevents its degradation. β-catenin then can accumulate in the cell and is transported to the nucleus to initiate transcription of Wnt target genes by binding to T-cell factor/lymphoid enhancer binding factor (TCF/LEF) transcription reporters. One or the Wnt target genes is AXIN2 [42]. Wnt signaling is regulated by many proteins, including Wnt antagonists sFRP proteins. The sFRP protein family consists of five members, sFRP1, sFRP2, sFRP3 (FRZB), sFRP4, and sFRP5. sFRP proteins share sequence similarity with the cysteine-rich domain found in the extracellular region of the Frizzled receptor, which is the Wnt binding site. Therefore, sFRPs are able to interact with Wnt proteins and disrupt the Wnt–Frizzled complex [52].

In glaucomatous TM cells, a Wnt signaling antagonist, secreted Frizzled-related protein 1 (sFRP1), is upregulated, and sFRP1 upregulation correlated with IOP elevation in both an organ culture and mice [34,53]. Therefore, it was thought that the upregulation of Wnt signaling could be the cause of the glaucomatous TM cells in open-angle glaucoma [53]. However, in our earlier study, using DEX-treated primary TM cells to model glaucomatous phenotypes, we found that a Wnt signaling inhibitor, but not a Wnt signaling activator, suppressed the DEX-induced glaucomatous phenotypes, suggesting that sFRP1 upregulation is instead a consequence and not the cause of initial Wnt signaling activation in DEX-treated TM cells [36].

To further evaluate the above notion, in this study, we aimed to investigate the association between Wnt activation and sFRP1 expression in TM cells treated with DEX. To achieve this, we examined *AXIN2* gene expression levels as the Wnt activation marker; although most Wnt signaling target genes are tissue specific or developmental stage specific, the *AXIN2* gene is a global transcription target [42]. In addition, to monitor the DEX-treated HTM cells, we examined the *MYOC* gene expression and myocilin protein levels in the cells since the upregulation of *myocilin* (*MYOC*) expression upon glucocorticoid challenge is a well-established hallmark of TM cells. Interaction of myocilin with γ-synuclein affects its secretion and aggregation [54]. Over a 10-day period, we observed a correlated temporal relationship between the mRNA expressions of *MYOC*, *AXIN2*, and *sFRP1* genes in DEX-treated TM cells. Our findings suggest that *sFRP1* upregulation could be a result of a negative feedback mechanism generated by stressed TM cells to suppress abnormal Wnt signaling activities. These results support the idea that a dynamic understanding of the TM response to glucocorticoids is necessary to develop effective therapeutic reagents.

## 2. Materials and Methods

*Primary human TM (HTM) cell culture*. Three independent primary cultures of HTM cells were isolated from HTM tissue explanted from corneoscleral rims of three donors ranging from 46 to 67 years of age. All corneoscleral rims used for this study were disease free and considered suitable for corneal transplantation. Corneoscleral rims were preserved in individual containers containing Optisol-GS at 4 °C, and HTM tissue was explanted before the transplantation expiry date.

To explant HTM tissue, corneoscleral rims were cut into quadrants using a blade; each piece was about 5 mm long. Forceps were used to gently remove the iris in order to expose the HTM. The HTM was carefully dissected from the sclerocorneal rim and washed thoroughly with 1X Dulbecco’s Phosphate-Buffered Saline without Calcium and without Magnesium (DPBS w/o Ca^++^ and Mg^++^) obtained from VWR/Avantor, Radnor, PA, USA. The HTM segments were then placed under a 22 mm × 22 mm glass coverslip (VWR/Avantor) in a 6-well plate and incubated for approximately 30 min at 37 °C, 5% CO_2_, to allow for the HTM tissue and coverslip to adhere to the well. Following incubation, Complete Dulbecco’s Modified Eagle Medium (DMEM-low glucose (1 g/L)) with GlutaMAX^TM^ (Thermo Fisher Scientific, Waltham, MA, USA), supplemented with 20% fetal bovine serum (FBS; VWR/Avantor) and 1% Antibiotic-Antimycotic (AA; Mediatech, Inc., Manassas, VA, USA), was added to the adhered HTM tissues in each well. These media were carefully replaced every 2 days until a monolayer of HTM cells formed and cells reached confluency. These cells were assigned HTM6 (46, M), HTM13 (67, M), and HTM15 (59, F).

Upon reaching confluency, typically at about 5 to 7 days, the coverslip was gently lifted, and HTM cells were harvested from wells and expanded in gelatin-coated flasks containing DMEM-low glucose (1 g/L) with GlutaMAX^TM^ supplemented with 10% FBS and 1% AA. Cells at passage 4 were utilized for this study. This study was performed in compliance with the requirements of the Declaration of Helsinki.

*Cell assay*. After reaching confluency at passage 3, HTM cells were harvested and seeded into T25 cm^2^ flasks per donor at 5.0 × 10^5^ cells per flask. Following a 24 h incubation at 37 °C, 5% CO_2_, HTM cells were treated with either Complete DMEM + 10% FBS and 1% AA, 100nM DEX (Millipore Sigma, Carlsbad, CA, USA), or 0.0001% dimethyl sulfoxide (DMSO) (Millipore Sigma). DMSO is considered the “vehicle” since DEX was dissolved in 100% DMSO for a total of two flasks per treatment group. Day 0 was considered after HTM cells were treated with either Complete DMEM + 10% FBS and 1% AA, 100 nM DEX, or 0.0001% DMSO and incubated at 37 °C, 5% CO_2_. Approximately every 24 h for 10 days, two flasks per treatment group were randomly chosen and harvested for RNA and protein isolation and analysis of *MYOC*, *AXIN2*, and *sFRP1* gene expression and myocilin protein expression, respectively. The standard error was generated from the duplicate measurements.

Conditioned medium was replaced for the remaining flasks with the respective treated medium in sequential order until day 10. HTM15 cells were experimented on separately, but the exact protocols used for HTM6 cells and HTM13 cells were followed for HTM15 cells. RNA and protein were isolated from harvested HTM6 and HTM13 cells for gene expression analysis and myocilin protein expression. For treated HTM15 cells, only RNA samples were collected for analysis.

*Quantitative reverse transcription PCR (RT-qPCR).* To determine *MYOC*, *AXIN2*, and *sFRP1* gene expression for DEX-treated HTM cells over a 10-day period, each day, HTM cells from each treatment group were harvested approximately 24 h apart for RT-qPCR measurements. Following harvest, treated HTM cells were pelleted by centrifuging for 3 min at 50× *g*. Total RNA was extracted from treated HTM cells using the Qiagen RNeasy mini kit (Qiagen, Valencia, CA, USA) as per the manufacturer’s protocol. The concentration of RNA was measured using the NanoDrop 2000 (Thermo Fisher), and the quality of the RNA was determined on the ratio of absorbance at 260 nm and 280 nm. Relative levels of mRNA encoding *MYOC*, *AXIN2*, and *sFRP1* were assessed using TaqMan real-time quantitative PCR. This was carried out using the Eppendorf Mastercycler Realplex2 (Eppendorf, Hauppauge, NY, USA) with the qScript XLT One-Step RT-qPCR ToughMix (Quanta Biosciences, Gaithersburg, MD, USA) and TaqMan primers for human *GAPDH* (Thermo Fisher, Hs02758991_g1), human *AXIN2* (Thermo Fisher, Hs00610344), human *sFRP1* (Thermo Fisher, Hs00610060), and human *MYOC* (Thermo Fisher, Hs00165345). The manufacturer’s protocol was followed for the RT-qPCR reaction. Gene expression was normalized against *GAPDH*. All reactions were carried out in triplicate as per the manufacturer’s protocol for the RT-qPCR reaction. The delta-delta Ct method was utilized to analyze relative changes in gene expression over the 10-day period.

*Western blot.* HTM cells from each treatment group were harvested approximately every 24 h for 10 days. In brief, the medium was aspirated, and samples were washed in DPBS, centrifuged for 3 min at 50× *g*, and then the supernatant was discarded. Pellets were lysed in RIPA buffer, which was supplemented with protease and phosphatase inhibitor cocktails (Thermo Fisher Scientific), and placed on ice. These samples were vortexed for 15 s every 10 min for 40 min followed by centrifugation at 4 °C at maximum speed (~100× *g*) for 10 min. The Pierce BCA assay (Thermo Fisher), together with a FilterMax F5 microplate reader (Molecular Devices, Sunnyvale, CA, USA), was used to determine protein concentrations. The sample concentration was adjusted to 20 μg/μL for each sample. After separating on 6–12% SDS-PAGE gels, the protein samples were transferred to PVDF membranes (Bio-Rad Laboratories, Hercules, CA, USA), followed by three washes with 1X PBST Wash Buffer for 5 min per wash at room temperature on a shaker. Then, at room temperature, the loaded membranes were blocked with Superblock (Bio-Rad Laboratories) and incubated for approximately 60 min. After incubation at 4 °C, the loaded membranes were incubated with primary anti-myocilin antibody (1:500) and anti-β-actin (1:2000) (Santa Cruz Biotechnology, Santa Cruz, CA, USA) overnight. The membranes were then washed 3 times with 1X PBST Wash Buffer for 5 min per wash at room temperature on a shaker, followed by treatment with anti-goat-HRP and anti-mouse-HPR secondary antibody (1:5000) for approximately 60 min at room temperature. The membranes were washed with 1X PBST Wash Buffer 3 times for 5 min per wash at room temperature on a shaker. Finally, ECL substrate (Bio-Rad Laboratories) was added to the membranes based on the manufacturer’s protocol, and the membranes were placed carefully into an 8″ × 11″ cassette lined with a transparent/clear liner. An X-ray film developer in a darkroom was used to expose and develop an ECL film, which was placed on top of the lined cassette in the darkroom to detect myocilin and β-actin (loading control) bands. ImageJ was used to quantify myocilin protein expression level.

*Statistical analysis.* One-way ANOVA within GraphPad Prism (GraphPad Software, San Diego, CA, USA) was used together with Dunnett’s correction to obtain the standard error of mean and significance (*p* < 0.05 was considered statistically significant).

## 3. Results

Based on published studies [55,56,57], we established a robust protocol to successfully isolate and culture HTM cells from fresh human TM tissues. In this study, HTM cells were isolated from the tissues of a 46-year-old male donor (HTM6), a 67-year-old male donor (HTM13), and a 59-year-old female donor (HTM15). Treatment of HTM cells with Complete DMEM, 100 nM DEX, or 0.0001% DMSO began at passage 4.

**Figure 1:**
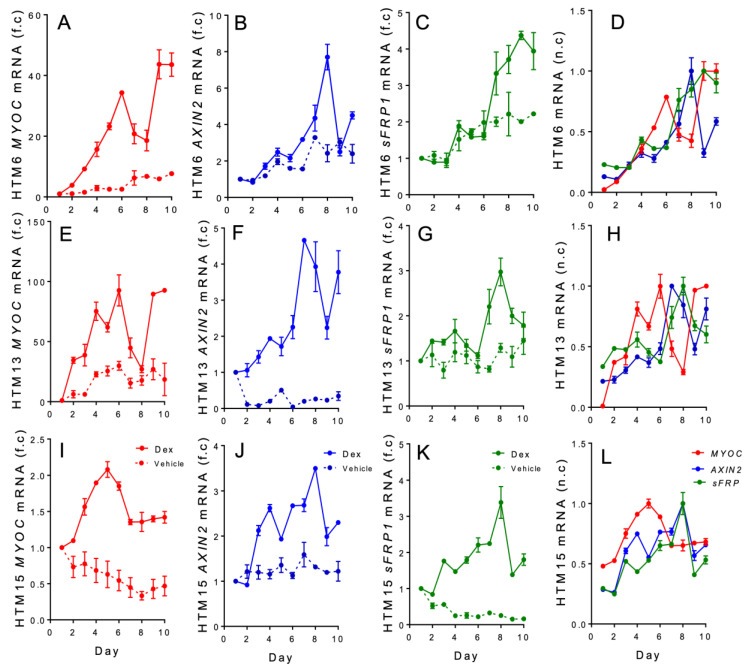
Dexamethasone mediated *MYOC*, *AXIN2*, and *sFRP1* gene expression in a sequential manner over a 10-day time course. The first row (**A**–**C**) shows the RT-qPCR analysis in fold change (f.c.) of *MYOC*, *AXIN2*, and *sFRP1* genes over 10 days in HTM6, followed by HTM13 (**E**–**G**) and HTM15 (**I**–**K**), respectively. All HTM cell batches were treated in duplicate with 0.0001% DMSO (vehicle) or with DEX (100 nM) for ten days. The standard error was generated from a duplicate measurement. The last column is an overlap of the n.c. (normalized change) of *MYOC*, *AXIN2*, and *sFRP1* gene expression over a 10-day course for the DEX-treated group (**D**,**H**,**L**). *MYOC* expression increased beginning on day 2 relative to the control (**A**,**E**,**I**). *AXIN2* (**B**,**F**,**J**) and *sFRP1* expression levels (**C**,**G**,**K**) were also enhanced in comparison to their respective control, as indicated by the fold change. A similar observation was made for the peak expression for all HTM cell batches used for this study. First, *MYOC* expression levels peaked, followed by *AXIN2* expression, and *sFRP1*, respectively (**D**,**H**,**L**). For HTM6, *MYOC* reached peak expression on day 6, *AXIN2* on day 8, and *sFRP1* peak expression on day 9 (**D**,**H**,**L**). For HTM13, *MYOC* reached peak expression on day 6, *AXIN2* on day 7, and *sFRP1* expression on day 8 (**D**,**H**,**L**). For HTM15, *MYOC* reached peak expression on day 5, *AXIN2* on day 8, and *sFRP1* expression on day 8 (**D**,**H**,**L**). Furthermore, as *sFRP1* levels were enhanced, *AXIN2* levels declined around day 8–9, and *MYOC* levels peaked once again around day 9–10 (**D**,**H**,**L**).

To determine the expression levels of the *MYOC*, *AXIN2*, and *sFRP1* genes, RT-qPCR was carried out over a 10-day treatment period in DEX-treated HTM cells. Our results demonstrate that *MYOC*, *AXIN2*, and *sFRP1* gene expression occurred in chronologic order (Figure 1A–L). *MYOC* expression peaked first (day 8 in HTM6 cells, day 6 in HTM13 cells, and day 5 in HTM15 cells), followed by *AXIN2* peak expression, which occurred on day 8 for HTM6 cells, day 7 for HTM13 cells, and day 8 for HTM15 cells. Furthermore, *sFRP1* expression levels peaked after *MYOC* and *AXIN2* (day 9 for HTM6 cells and day 8 for HTM13 and HTM15 cells). Moreover, for all three HTM cell cultures, when *sFRP1* expression levels increased, *AXIN2* levels declined around days 8–9, and *MYOC* levels peaked once again around days 9–10. These findings regarding *MYOC* expression are consistent with those of previous studies that showed that DEX induces the upregulation of *MYOC* mRNA in a time-dependent manner [58,59].

**Figure 2:**
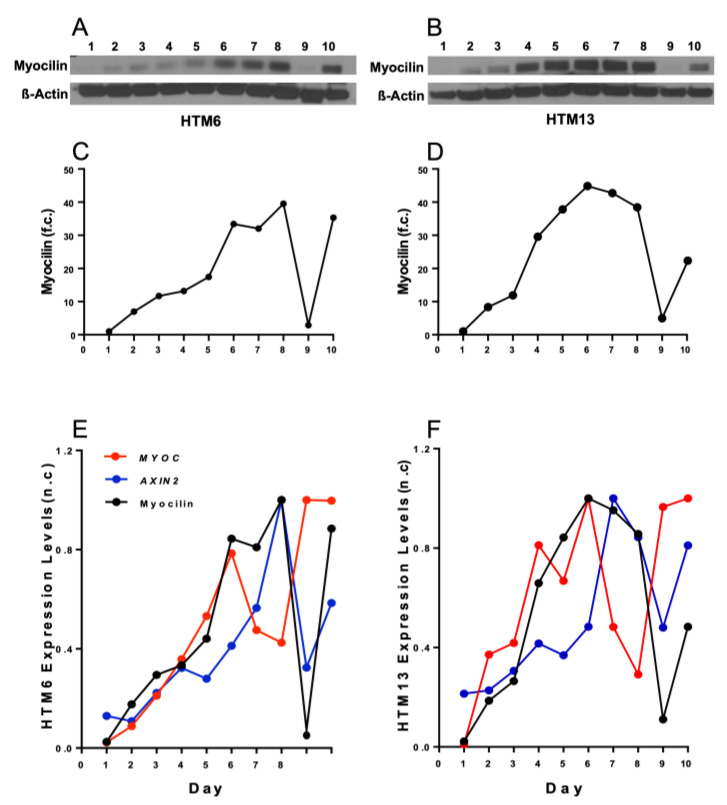
Myocilin protein expression of DEX-treated HTM cells over 10-day time course. Western blot analysis showed the 10-day study of DEX-induced myocilin protein levels for cell batches HTM6 and HTM13. HTM cells were treated with DEX (100 nM) for 10 days. Each day, two flasks were randomly selected and harvested to isolate cytoplasmic fractions. These cytoplasmic fractions were analyzed by Western blot with antibodies against myocilin and β-actin. Β-actin was used as a control and for normalization. Images of the gels are presented in (**A**,**B**). The background contrast was adjusted, and blots were quantified for fold change (f.c.) using the ImageJ software. Western blot analysis showed that myocilin protein levels began increasing on day 2, and a similar observation was made for both batches. Myocilin protein levels first peaked on day 6 for HTM6 and for HTM13 (**C**,**D**). Myocilin protein levels dropped on day 9 for both DEX-treated HTM cell batches (**C**,**D**). Overlay of quantitative qPCR and Western blot analysis, as shown by normalized change (n.c.), indicates that there was a correlation between *AXIN2* and myocilin expression. Overlay of DEX-induced *MYOC* and *AXIN2* gene expression and myocilin protein expression of HTM6 and HTM13 over 10-day time course (**E**,**F**). Over 10 days, *AXIN2* and *MYOC* expression was enhanced; however, there was an increase in MYOC expression on day 8 and day 9 and a sharp decline of *AXIN2* gene expression and myocilin protein expression on day 9.

Additionally, myocilin protein levels in the DEX-treated HTM6 and HTM13 cells were analyzed using Western blot analysis (Figure 2). Although myocilin protein levels also increased in a time-dependent manner during DEX treatment, the increase in myocilin protein trailed the increase of the *MYOC* mRNA levels. Nevertheless, it seems that the myocilin protein levels were synchronized with the mRNA expression of *AXIN2* or *sFRP1*. When the expression of *AXIN2* and *sFRP1* reached its highest levels, myocilin protein expression declined (Figure 2E,F).

## 4. Discussion

In this study, we identified a causal nexus between the glaucoma-associated gene *MYOC* and Wnt signaling components *AXIN2* and *sFRP1* in DEX-treated HTM cells. We observed that the expression levels of *MYOC* and Wnt signaling genes *AXIN2* and *sFRP1* were augmented in a consecutive manner at different time points in all the DEX-treated HTM cells derived from three different donors. Specifically, *MYOC* expression peaked first, followed by the active Wnt signaling activity marker *AXIN2* and, lastly, Wnt signaling inhibitor *sFRP1*.

Increased *MYOC* expression upon glucocorticoid challenge is a well-established hallmark of TM cells [56,57]. Myocilin can be induced by various stimuli in HTM cells [60,61]. Although the exact function of myocilin in the TM remains unclear, *MYOC* overexpression may indicate abnormalities occurring in the HTM. For instance, oxidative stress and TM cell stretch have been shown to induce myocilin levels in HTM cells. Myocilin potentially modulates Wnt signaling pathways through targeting Wnt ligand receptors, Fzds, and Wnt inhibitors, such as sFRP1 and sFRP3 [62]. Indeed, overexpression of myocilin in the TM cells can activate Wnt signaling [63]. Therefore, it is conceivable that the elevation of *MYOC* expression induced by the DEX treatment may activate Wnt signaling in the TM cells [36]. As Wnt signaling is a key player in maintaining TM tissue homeostasis [64], we previously proposed that, in DEX-treated TM cells, Wnt signaling activity may function as a defense mechanism against the various stressors induced by DEX treatment [36]. Consistent with this notion, we found in this study that expressions of the *MYOC* and *AXIN2* genes, a well-documented indicator of Wnt signaling activation [42], were both upregulated in the HTM cells within the first three days of DEX treatment, yet *MYOC* levels peaked before *AXIN2* levels peaked. Moreover, *AXIN2* expression was closely synchronized with the myocilin protein level in the DEX-treated TM cell, suggesting that myocilin may play a role in activating Wnt signaling in TM cells. Although activated Wnt signaling may play a key role in maintaining TM tissue homeostasis, Wnt signaling exhibits pleiotropic roles. Some of the phenotypes linked to steroid-induced glaucoma could be attributed to the downstream effects of activated Wnt signaling molecules. Indeed, it has been reported that Wnt activation is present in the TM pathobiology [37]. Therefore, to maintain the homeostatic state of the TM tissue, the activated Wnt signaling must be “reset” during prolonged steroid treatment.

Studies have indicated that a Wnt signaling inhibitor, sFRP1, is overexpressed in DEX-treated HTM cells and glaucomatous tissue [53,65]. Continuously suppressing Wnt signaling via Wnt antagonists, such as sFRP1 and Dkk1, was shown to damage TM tissue in mice models [53,65]. In addition, a mouse model showed that intravitreal injection of a viral vector encoding sFRP1 resulted in significantly elevated IOP [53]. Furthermore, an enhanced level of sFRP1 was linked to decreased outflow facility in perfusion-cultured human anterior segments [66]. Therefore, sFRP1 may act as a negative feedback mechanism generated by “stressed” TM cells to suppress abnormal Wnt signaling activities. Such a feedback mechanism has been reported in cardiomyocyte differentiation [67]. Indeed, our study revealed that the expression levels of *sFRP1* were increased within 24 h following the overexpression of Wnt signaling levels. Additionally, *MYOC* expression levels showed a subsequent increase approximately 24 h following peak *sFRP1* levels. Therefore, we propose that *sFRP1* levels are significantly upregulated due to abnormal Wnt signaling in DEX-treated HTM cells.

If the upregulation of *MYOC* expression induces Wnt activation in DEX-treated TM cells, to control Wnt activity, the expression of *MYOC* has to be regulated as well. Indeed, in our study, we found that during the prolonged DEX treatment in the cells, the *MYOC* mRNA level did not continuously increase; instead, it started to decrease after peaking around day six, and the concentration of myocilin protein also peaked around day seven. Consistent with our study, several early studies showed that the expression of both *MYOC* mRNA and myocilin protein in HTM cells that have received prolonged DEX treatment is not continuously upregulated [7,58,59]. Our observations suggest that the expression of *MYOC* in prolonged DEX-treated TM cells can still be regulated, even at a much higher level. It is known that TM cells’ responsiveness to glucocorticoids is greatly determined by the TM glucocorticoid receptor ratio of GRα (glucocorticoid receptor alpha) to GRβ (glucocorticoid receptor β), with GRβ being the negative regulator [68,69]. However, because *MYOC* expression is induced within the second day of DEX treatment, the upregulation of *MYOC* expression is considered a secondary response to the DEX treatment [58,59]. Therefore, we speculate that many cellular events stimulated by the DEX treatment have the potential to contribute to the regulation of MYOC expression; Wnt signaling is likely to be one of those events. Previously, we showed that a Wnt signaling inhibitor reduces *MYOC* expression in DEX-treated HTM cells [36]. It was currently reported that a GSK-3β inhibitor, which can activate canonical Wnt signaling pathway, reduces the basal expression of MYOC in HTM cells [70]. The current study showed that the downregulation of *MYOC* expression correlates with Wnt signaling activity and *sFRP1* expression. However, our data do not provide enough resolution to determine whether Wnt activation or inhibition is responsible for the downregulation of *MYOC* expression in DEX-treated HTM cells.

## 5. Conclusions

Abnormal activation of the Wnt signaling pathway has also been clearly implicated in glaucomatous TM phenotypes [37,53,66,71]. Our study demonstrated a time-dependent upregulation of MYOC, Wnt signaling activity, and the expression of a Wnt signaling inhibitor sFRP1 in human TM cells under DEX treatment for ten days. While we observed that Wnt signaling is activated in TM cells in response to external stress signals, such as DEX treatment, the activated Wnt signaling in the TM cells is likely to have multiple downstream effects. While some of these effects may be beneficial in maintaining homeostasis, others may be unfavorable. Consequently, after the activation of Wnt signaling in the TM cells, the mechanism that suppresses Wnt signaling is also activated in response to these harmful events. Thus, DEX-treated TM cells continually adjust and readjust their responses to external stress.

Therapeutic use of glucocorticoids can cause an elevation of IOP [15,16,72], leading to optic nerve damage, resulting in steroid-induced glaucoma [4,18]. Therefore, it is desirable to develop reagents that can be used to reduce or eliminate the IOP elevation during glucocorticoid therapy. Nevertheless, to develop such reagents, the dynamic nature of the TM cell’s response to glucocorticoids needs to be fully considered.

## Data Availability

Not applicable.
